# Compartmental structures used in modeling COVID-19: a scoping review

**DOI:** 10.1186/s40249-022-01001-y

**Published:** 2022-06-21

**Authors:** Lingcai Kong, Mengwei Duan, Jin Shi, Jie Hong, Zhaorui Chang, Zhijie Zhang

**Affiliations:** 1 Department of Mathematics and Physics, North China Electric Power University, Baoding, 071003 China; 2 Department of Epidemiology and Health Statistics, Fudan University, Shanghai, 200032 China; 3 Division of Infectious Disease, Key Laboratory of Surveillance and Early-Warning on Infectious Disease, Chinese Center for Disease Control and Prevention, Beijing, China

**Keywords:** COVID-19, Dynamic model, Compartment, Epidemic model

## Abstract

**Background:**

The coronavirus disease 2019 (COVID-19) epidemic, considered as the worst global public health event in nearly a century, has severely affected more than 200 countries and regions around the world. To effectively prevent and control the epidemic, researchers have widely employed dynamic models to predict and simulate the epidemic’s development, understand the spread rule, evaluate the effects of intervention measures, inform vaccination strategies, and assist in the formulation of prevention and control measures. In this review, we aimed to sort out the compartmental structures used in COVID-19 dynamic models and provide reference for the dynamic modeling for COVID-19 and other infectious diseases in the future.

**Main text:**

A scoping review on the compartmental structures used in modeling COVID-19 was conducted. In this scoping review, 241 research articles published before May 14, 2021 were analyzed to better understand the model types and compartmental structures used in modeling COVID-19. Three types of dynamics models were analyzed: compartment models expanded based on susceptible-exposed-infected-recovered (SEIR) model, meta-population models, and agent-based models. The expanded compartments based on SEIR model are mainly according to the COVID-19 transmission characteristics, public health interventions, and age structure. The meta-population models and the agent-based models, as a trade-off for more complex model structures, basic susceptible-exposed-infected-recovered or simply expanded compartmental structures were generally adopted.

**Conclusion:**

There has been a great deal of models to understand the spread of COVID-19, and to help prevention and control strategies. Researchers build compartments according to actual situation, research objectives and complexity of models used. As the COVID-19 epidemic remains uncertain and poses a major challenge to humans, researchers still need dynamic models as the main tool to predict dynamics, evaluate intervention effects, and provide scientific evidence for the development of prevention and control strategies. The compartmental structures reviewed in this study provide guidance for future modeling for COVID-19, and also offer recommendations for the dynamic modeling of other infectious diseases.

**Graphical Abstract:**

**Supplementary Information:**

The online version contains supplementary material available at 10.1186/s40249-022-01001-y.

## Background

After emerging in late 2019, the coronavirus disease 2019 (COVID-19) pandemic has affected more than 200 countries and territories, with more than 507.5 million confirmed cases and over 6.22 million deaths reported globally as of April 25, 2022 [1]. The speed, scope, and difficulty of prevention and control of the epidemic are unprecedented. It was declared a “global pandemic” by the World Health Organization (WHO) on March 11, 2020 [2]. The pandemic has not only posed a serious threat to human health but has also had profound consequences on society, economy, environment, public psychology, and so on [3]. In the early stages, as a newly emerging infectious disease, the epidemiological characteristics, transmission mechanisms, and clinical features of COVID-19 were not clear. At this time, due to the ability to combine expert advice and the limited data needed, dynamic models are being widely used to predict the dynamic trends, intensity, and temporal and spatial dynamic processes of the epidemic and to evaluate the potential impact and effectiveness of candidate prevention and control measures. Therefore, this has played an important role in allocating medical and health resources reasonably, determining effective prevention and control measures, and formulating strategies for the resumption of work and production in the early stages of the epidemic.

After almost two years of COVID-19, the scientific community has more in-depth research and a greater understanding of its epidemiology, characteristics, clinical manifestations, and other aspects. Due to the distinct incubation period of COVID-19, early studies using the susceptible-infected-recovered (SIR) model and its extension may be inaccurate, while the susceptible-exposed-infected-recovered (SEIR) model and its extension are more appropriate. As the characteristics of the COVID-19 epidemic have been revealed, and various non-pharmaceutical interventions (NPIs) have been applied, the compartmental structures of COVID-19 dynamic models have become increasingly rich and complex to reflect the true transmission dynamics of the epidemic to the greatest extent. A reasonable compartmental structure is critical for dynamic modeling; therefore, it is vital to review the compartmental structures of the COVID-19 dynamic models.

In this review, we analyzed the compartmental structures used in COVID-19 dynamic models, including the SEIR-based expanded models, meta-population models, and agent-based models, hoping to provide an important reference for the dynamic modeling for COVID-19 and other infectious diseases in the future.

## Methods

### Database searches

To conduct the search, we entered “COVID-19” in the title field, and “dynamics model” related fields in the abstract field. The search strategy was as follows: (COVID-19 + Novel Coronavirus + 2019-nCOV + nCOV-19 + SARS-CoV-2) * (SIR + SEIR + SIRQ + SEIRQ + "the reproductive number" + "stochastic model" + " deterministic model" + "compartment model" + "dynamics model" + "mathematical model" + "mechanism model" + "meta-population model" + "agent-based model" + "individual model" + "epidemic model" + "simulation model").

The searches, conducted on May 14, 2021, yielded 4499 records across PubMed, ScienceDirect, and Web of Science, and 405 records (in Chinese) across CNKI and Wanfang Database [4, 5].

### Record screening

**Fig. 1:**
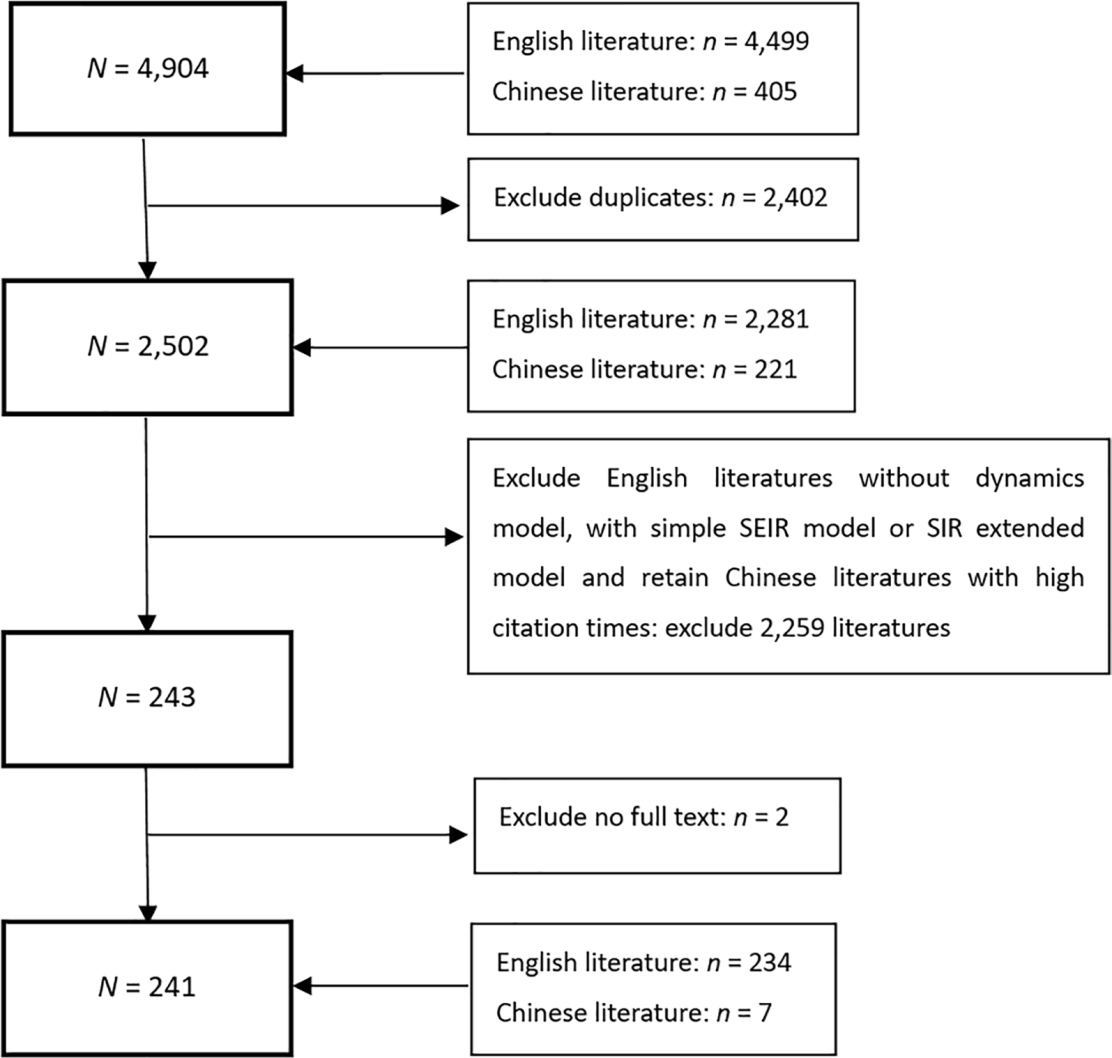
Literature search process, including inclusion and exclusion criteria for articles screen

The inclusion criteria were as follows: (1) the literature language was either English or Chinese; (2) focus on COVID-19; (3) the compartmental structures of the dynamic model were described in detail; (4) literature with high reference value determined by internal expert discussion (mainly for papers in Chinese, based on the core journals of Peking University). The exclusion criteria were as follows: (1) repeated articles; (2) no dynamic model was established, SIR or its extension, or only a basic SEIR model was used; (3) full text unavailable. According to the above criteria, 234 English and 7 Chinese references were included in this review. The screening process is represented in Fig. 1.

## Results

Due to incubation period of COVID-19, the SEIR model should theoretically be used as the basis to construct compartment models. Focusing on the compartmental structures used, this review analyzed the expanded compartment models based on the SEIR model, meta-population models, and agent-based models.

### Expanded models based on SEIR

**Fig. 2:**
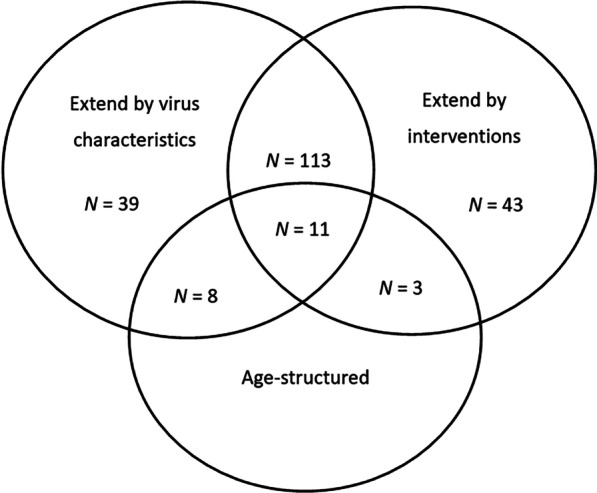
Main reasons to expand the compartments of the SEIR model

The main reasons to expand the compartments of the SEIR model include the following (Fig. 2):

1. the COVID-19 characteristics, such as asymptomatically infected, death, further subdividing the infected compartment by disease status, etc.
2. public health interventions, such as hospitalization, isolation, quarantine, etc.
3. age structure.
4. integrating the above reasons.

### Expand compartments according to COVID-19 characteristics

Understanding the transmission mechanism and viral characteristics of COVID-19 is critical to control its spread. COVID-19 is primarily transmitted from person to person through respiratory droplets released when someone with COVID-19 sneezes, coughs, or talks. People can also be infected by touching objects contaminated with live viruses or by touching mucous membranes, such as the mouth, nose, and eyes after exposure to a contaminated environment [6]. Infected cases can be divided into symptomatic patients and asymptomatic patients according to the presence or absence of clinical symptoms; the symptomatic patients can be further divided into mild patients, severe patients, and critically ill patients (ICU patients), according to the severity of their symptoms. Therefore, to model the transmission dynamics, researchers expanded the compartments according to these divisions [7–9]. A proportion of those infected died, and the death rate of severe patients was higher than that of mild patients [10]. Thus, the death compartment (D) is included in some models. In addition, some studies added a compartment to account for the possible transmission to people via the live virus in contaminated environments [11–13]. A summary of the expanded compartments according to virus characteristics is presented in Table 1 (complete compartmental structures and meaning are shown in Additional file 1).

**Table 1:** Summary of the expanded compartments according to virus characteristics of COVID-19

Expanded compartments	Interpretation	References
A	Asymptomatic	[14]
I_m_, I_s_	Mild (I_m_)/ severe (I_s_) symptom	[7]
I_c_	Critical	[9]
I_p_, I_c_, I_s_	Preclinical (I_p_), clinical (I_c_), subclinical infection (I_s_)^a^	[15]
P	Pre-symptomatic	[16]
D	Dead	[17–19]
B	Live virus in environment	[11]

^a^Subclinical infected: infected persons with mild or no symptoms; clinical infected: infected persons with obvious symptoms.

### Expand compartments according to public health interventions

Before the COVID-19 vaccination became available, countries worldwide adopted various NPIs, including border closure, active contact tracing, testing for isolating cases, quarantining suspected cases, wearing face masks, social distancing, school and/or workplace closure, travel restrictions, etc., in an effort to mitigate and contain the transmission. To simulate the true transmission dynamics of COVID-19 to the greatest extent and to assess the effects of different NPIs, researchers added compartments reflecting NPIs. These NPIs can be divided into the following two categories according to the target population of interventions:

1. Protect the susceptible population. The measures include restricting the activities of the susceptible population in medium- and high-risk areas [20, 21]; changing behaviors of the susceptible population through propaganda and education on epidemic prevention and control [22–24], requiring or encouraging people to wear face masks, maintain social distancing, wash hands frequently, reduce gatherings, and so on. These measures were meant to reduce the risk of exposure to COVID-19.
2. Track and isolate infected or suspected infected individuals and close contacts. The measures include isolating and hospitalizing infected individuals [25] and quarantining close contacts and suspected cases [26]. These measures can effectively reduce contact between the infectious and susceptible population, thereby reducing transmission rates. Considering these measures, researchers added corresponding isolation, quarantine, and hospitalization compartments based upon a basic SEIR model. The isolation compartments can be subdivided according to close contacts, suspected cases, and different types of infected persons. Hospitalized patients have been further subdivided according to their severity of symptoms, as indicated below.

In addition to the above two categories of NPIs, active nucleic acid testing and timely reporting of cases [27] were also important prevention and control measures. On the one hand, testing for close contacts, sub-close contacts (close contacts of close contacts), and suspected cases, testing for all people in high-risk areas, and testing for people out from high-risk areas can detect infected cases and take quarantine or other measures to stop the transmission; on the other hand, notification of confirmed cases and their travel paths can also improve the self-protective consciousness of the susceptible population and therefore reduce the risk of infection.

The expanded compartments reflecting public health interventions are presented in Table 2 (see Additional file 2 for the complete structures and meaning of the compartments).

**Table 2:** Expanded compartments based on public health interventions

Intervention	Expanded compartments	Interpretation	References
Categorize the susceptible	S_p_	Protected susceptible^a^	[20]
	S_c_	Confined susceptible^a^	[21]
	S_r_	Behavior changed susceptible^a^	[24]
	M, U	Masked/unmasked humans	[23]
Hospitalization/quarantine	H	Hospitalized infected	[25]
	Q	Quarantine	[28]
	S_q_, E_q_	Quarantined susceptible (S_q_), quarantined exposed (E_q_)	[26]
Active nucleic acid testing and notification of confirmed cases	M	Missed cases	[29]
	Q_1_, Q_2_	Suspected population under home quarantine (Q_1_), medical quarantine population of confirmed cases (Q_2_)	[30]
	I_r_, I_u_	Tested infected individuals (I_r_), non-tested infected individuals (I_u_)	[27]
	I_1_, I_2_	Infectious people with timely diagnosis(I_1_), delayed diagnosis (I_2_)	[31]

^a^Protected, confined, and behavior changed susceptible people are less likely to be infected than ordinary susceptible people

### Expanded compartments based on age structure

Some studies considered the heterogeneity of the population, such as different contact rates among people, infection rates for different individuals, the protection awareness of people with different occupations and at different ages, and different development processes of the disease due to different physical fitness levels after infection. Additionally, the vaccination has certain regulations and priorities for different ages.

Table 3 summarizes age groups and compartmental structures in the model in relation to age structure. Additional file 3 indicates the complete compartmental structures and age groups.

**Table 3:** Age groups and compartmental structures of dynamic models considering age structure

Age group, years	Compartmental structures	Interpretation	References
0–15, 15–29, 30–59, 59 +	S_i_,E_i_,A_i_,M_i_,H_i_,C_i_,R_i_	Susceptible (S_i_), exposed (E_i_), asymptomatic (A_i_), mild (M_i_), severe (H_i_), critical (C_i_), recovered (R_i_) in age group i	[32]
0–9, 10–19, …, 70–79, 80 +	S_i_E_i_A_i_I_i_H_i_R_i_D_i_	Susceptible (S_i_), latently infected (E_i_), asymptomatic infectious (A_i_), infectious individuals with symptoms/clinically ill (I_i_), hospitalized patients (H_i_), recovered (R_i_), death due to disease (D_i_) in group i	[33]
0–14, 15–49, 50–69, 70–80, 80 +	S_i_E_i_L_i_I_i_R_i_T_pi_A_si_S_si_S_vi_C_ri_R_di_D_i_	Susceptible (S_i_), exposed (E_i_), post latency (L_i_), infectious (I_i_), undocumented recovered (R_i_), tested positive (T_pi_), asymptomatic (A_si_), symptomatic (S_si_), severe (S_vi_), critical (C_ri_), dead (D_i_), documented recovered (R_di_) in age group i	[34]
0–14, 15–49, 50–69, 70 +	S_ij_E_ij_I_ij_Q_ij_H_ij_R_ij_D_ij_	Susceptible (S_ij_), exposed (E_ij_), presymptomatic (Ip_ij_), mild to moderate (I_mij_), severe (I_sij_), quarantined and exposed (Q_Eij_), pre-symptomatic and isolated (Q_Ipij_), mild to moderate and isolated (Q_Imij_), severe and isolated (Q_Isij_), isolated (Q_ij_), admitted to hospital (H_ij_), pre-ICU (P_ICUij_), ICU (H_ICUij_), recovered (R_ij_), dead (D_ij_) in age group I and health status j	[35]
0–10, 10–20, …, 60–70, 70 +	S_i_V_i_E_i_E_vi_A_i_A_vi_I_i_Q_i_R_i_R_vi_D_i_	Susceptible (Si), vaccinated (V_i_), exposed (Ei), exposed and vaccinated (E_Vi_), asymptomatic (A_i_), asymptomatic and vaccinated (A_Vi_), symptomatic (I_i_), isolated (Q_i_), recovered (R_i_), recovered and vaccinated (R_Vi_), death (D_i_) in age group i	[36]

### Integrating virus characteristics and interventions

More studies have considered both virus characteristics and intervention measures when constructing dynamic models, including subdividing compartments into more detailed levels according to whether the individual has been inspected [37], discovered [38], and reported [39]. These compartmental structures reflect better the actual situation of intervention. For example, some studies have modeled intervention measures for patients with different infection status, which were common in reality: isolating asymptomatic infections at home, admitting symptomatic infections to hospital [40], and admitting severe patients to ICU for treatment [41], etc. The recovered individuals were also divided into different compartments according to whether they had been detected or not, symptomatic or asymptomatic [37]. With the successful development of COVID-19 vaccination, researchers added the vaccination compartment to evaluate its effectiveness and to develop immunization programs [42]. The expanded compartments are shown in Table 4 (see Additional file 4 for the complete structures).

**Table 4:** Expanded compartments that considered both virus characteristics and interventions

Interventions	Expansion compartments	Interpretation	References
Social distancing, wearing masks, washing hands, etc	U	Unsusceptible ^a^(U)	[43]
Quarantined at home/hospitalization	S_T_	Susceptible persons removed from isolation ^b^(S_T_)	[44]
	I_2_	Infectious after receiving ineffective treatment (I_2_)	[45]
	Q, D	Home quarantined individuals (Q), diagnosed individuals who are being treated and isolated (D)	[46]
	I_a_, I_s_, Q_a_, Q_s_, R_u_, R_a_, R_s_	Undetected asymptomatic infectious (I_a_), undetected symptomatic infectious (I_s_), detected and quarantined asymptomatic (Q_a_), detected and quarantined symptomatic (Q_s_), undetected recovered asymptomatic (R_u_), recovered detected asymptomatic (R_a_), recovered detected symptomatic (R_s_)	[37]
	W_1_, R_1_, D_1_, W_2_, R_2_, D_2_	Hospitalized that never require an intensive care bed (W_1_), recovered from non-ICU (R_1_), deaths from non-ICU (D_1_), hospitalized that require an intensive care bed (W_2_), recovered from ICU (R_2_), deaths from ICU (D_2_)	[41]
	H_1_, H_2_	Confirmed cases who are quarantined at home (H_1_), confirmed cases who are hospitalized (H_2_)	[40]
	I_p_, I_c_	Primarily infected (I_p_), chronically infected (I_c_)^c^	[47]
Vaccination, testing or contact tracing	A_C_	Contact traced asymptomatic (A_C_)	[48]
	E_u_, E_d_	Undetected exposed (E_u_), detected exposed (E_d_)	[38]
	S_u_, S_v_	Unvaccinated susceptible (S_u_), vaccinated susceptible (S_v_)	[42]

^a^Unsusceptible: a susceptible person can become unsusceptible due to factors such as the use of facemasks, hand washing, and SD (social distance)

^b^Susceptible persons removed from isolation (S_T_): isolated susceptible individuals, after a period, are released from isolation and transferred to compartment S_T_

^c^Primarily infected: individuals that remain infectious within the reported duration of the infectious period after the incubation period; chronically infected: individuals that are less infectious but remain infectious and may be diagnosed for a longer duration

### Meta-population model

Meta-population models contain several subpopulations, each of them representing a spatial area from a country or a city, to a school or a family, to investigate interactions and movements among different subpopulations. A whole compartmental structure was conducted in each subpopulation to distinguish different populations and the movement of people among different subpopulations interacting with the whole population. Therefore, the meta-population models can be regarded as a combination of classic compartment models with network models. The compartments of the former have been analyzed above, and the latter is related to network analysis, with a relatively simple structure. The key to meta-population models lies in how to accurately describe the network to reflect reality and therefore to describe the movements of individuals among subpopulations, as well as their influence on the entire population.

By considering the contact heterogeneity and movements among subpopulations, meta-population models partially overcome the shortcomings of homogeneous mixing that traditional compartment models have typically assumed. They can also analyze the spatiotemporal dynamic process of infectious diseases on a relatively large spatial scale. Readers interested in this can refer to relevant studies [49].

Most of the compartmental structures of current meta-population models for COVID-19 were relatively simple. For example, Chang et al. established a SEIR meta-population model to identify high-risk areas of disease transmission and evaluated the potential influence of local travel restrictions in Taiwan, China with the population flow data [50]. Chan et al. employed a SEIR meta-population model combined with a dynamic mobile network to describe the prevalence of COVID-19 in 10 major cities of the United States [51]. Chinazzi et al. established a global SEIR meta-population model based on air flight networks to analyze the impact of travel restrictions on the spread of COVID-19 [52]. This type of model generally has a simple compartmental structure and can be found in previous summaries; we will not list them separately.

### Agent-based model

Traditional compartment models typically assume that the population were homogeneously mixing, as do the subpopulations in meta-population models; therefore, they cannot describe the heterogeneity between individuals. Agent-based models (ABMs) are simulation models in which entities (referred to as agents) interact with each other, considering the individual’s demographics, social environment, and natural environment. ABMs consist of a series of interaction rules to make agents regularly move between different places; therefore, they can simulate the real spatiotemporal spread of infectious diseases optimally in small-scale spaces from a microscopic level, and they can provide scientific evidence for the implementation of precise prevention and control. Therefore, ABMs can be regarded as an integration of dynamic models and interaction rules. Considering the complex interactions between individuals in a heterogeneous population, Hoertel et al. established a stochastic agent-based microsimulation model to examine the potential impact of post-lockdown measures in France [53]; Aleta et al. used mobility and demographic data in the Boston metropolitan area to build a detailed agent-based model, demonstrating the importance of testing and tracing in the context of relaxed social distancing [54]. However, ABMs need detailed data and heavy computation, especially with a large number of agents and complex rules; the results were influenced greatly by initial values and interaction rules. Therefore, in the published ABM-related research, the compartmental structures were relatively simple and not as complicated as the expanded compartments summarized above (Some compartmental structures applied by ABM were shown in Additional file 5).

## Discussion

Dynamic models are important tools in the study of infectious disease. By constructing compartments according to infection states and related interventions and simulating transformation among different compartments, they reflect the transmission process of diseases. The models can predict the development trends of the diseases and reflect the potential transmission process and evaluate the effectiveness of various intervention measures; therefore, they play a key role in creating prevention and control measures. Establishing appropriate compartmental structures is the basis and premise of making dynamic models work. We divided the dynamics models of COVID-19 into three categories, expanded compartment models based on SEIR model, meta-population models, and ABMs and review their compartmental structures accordingly, hoping to provide a reference for modeling COVID-19 in different scenarios and provide scientific guidance for modeling research on other diseases.

After reviewing current COVID-19 modeling studies, we found that the SEIR-based expanded models consider the latent period of the virus and expand the compartments according to the infection status and measures, such as tracking, diagnosis, and isolation. With the normalization of epidemic prevention and control, methods to reflect the intensity and time of various intervention measures in the model are still worthy to study. All the three types of models have their own characteristics and application scope, with different requirements for data. Traditional compartmental models generally assume homogeneous mixing of people, which is not realistic in the real world. However, they are simple in mathematical form, easy to analyze, low data requirements, and easy to apply. Meta-population models take population movements into account, therefore, are suitable for studying the spread of infectious diseases between different countries/regions. ABMs integrated heterogeneity of agents, make them be available for building more realistic models and provide accurate support for making decisions about the prevention and control of infectious diseases. These two latter types of models are more in line with real world, while need more parameters and complex rules of individual interaction, and require a greater demand on calculation resources. In practical application, appropriate models should be selected according to different research objectives, specific problems and data availability.

As time transpires, the COVID-19 epidemic, interventions, and people’s responses exhibit different characteristics: due to the effective implementation of early NPIs, subsequent successful development and mass vaccinations against COVID-19, the continued emergence of variant strains, and the overall acceleration of variation, people's productivity and lives are gradually returning to normal, and various sports events and public activities that had been cancelled or postponed due to the epidemic have been resuming. These characteristics render the development trend of the COVID-19 epidemic more complicated and unpredictable. In addition, adjusting the model according to changes in people’s awareness of prevention is also an issue that warrants close attention. At the same time, we also need to consider the possibility of reinfection among the vaccinated population, changes in the transmission capacity of mutant viruses, and the frequency and time points of implementation of various measures in the models, which can help us predict the epidemic, evaluate the effectiveness of prevention and control measures, and formulate prevention and control strategies more accurately, so as to ensure the safe and smooth development and recovery of public events and sports events as well as provide a scientific basis to meet the new demands in the pandemic.

## Conclusions

We comprehensively reviewed the current COVID-19 dynamic models and mainly analyzed the expanded models based on the SEIR model, meta-population models, and ABMs. We found that the SEIR-based expanded models were created mainly according to the COVID-19 characteristics, NPIs, and the age structure of the population, which have been relatively mature and comprehensive, but further research is needed with the vaccination and the emergence of mutant strains. The meta-population models and the ABMs usually adopt a relatively basic or simple extended compartmental structure, which can be the focus of future research. Unsolved problems such as how and when to implement prevention and control measures accurately still require the help of dynamic models, for which the compartmental structures are of primary importance. This study can provide an important reference for the construction of compartments in future COVID-19 modeling. This may be applicable, for example, when constructing a reasonable dynamic model to simulate and evaluate the effects of different interventions (and their different implementation intensity and frequency) on the prevention and control of COVID-19 during large-scale sports events, or when lifting NPIs stepwise, etc. For the modeling of other respiratory infectious diseases, it also offers important guidance value.

## Supplementary Information

**Additional file 1.** Expanded compartmental structures based on SEIR according to virus characteristics of COVID-19.

**Additional file 2.** Expanded compartmental structures based on SEIR according to public health interventions.

**Additional file 3.** Compartmental structures of compartmental models for COVID-19 considered age structures.

**Additional file 4.** Compartmental structures that considered both virus characteristics and interventions.

**Additional file 5.** Compartmental structures of agent-based models for COVID-19.

## Data Availability

Not applicable.

## Abbreviations

COVID-19: The coronavirus disease 2019
WHO: World Health Organization
SIR: Susceptible-infected-recovered
SEIR: Susceptible-exposed-infected-recovered
NPIs: Non-pharmaceutical interventions
ABMs: Agent-based models
