# Supplementary material for: Compartmental structures used in modeling COVID-19: a scoping review
Source: Infect Dis Poverty. 2022 Jun 21;11:72. doi: 10.1186/s40249-022-01001-y (PMC9209832; doi:10.1186/s40249-022-01001-y)
Supplement: Supplementary file 1 — Additional file 1. Expanded compartmental structures based on SEIR according to virus characteristics of COVID-19. [file 40249_2022_1001_MOESM1_ESM.docx]

Table Expanded compartmental structures based on SEIR according to virus characteristics of COVID-19.

| **Model structure** | **Interpretation** | **Reference** |
| --- | --- | --- |
| **SEIRD** | susceptible (S), exposed (E), infectious (I), recovered (R), dead (D) | [[1-10](#_ENREF_1)] |
| **SEPIR** | susceptible (S), exposed (E), pre-symptomatic (P), infectious (I), recovered (R) | [[11](#_ENREF_11)] |
| **SETAIR** | susceptible (S), exposed (E), transitional^1^ (T), asymptomatic (A), infectious (I), recovered (R) | [[12](#_ENREF_12)] |
| **SEAIR, SEAIRD** | susceptible (S), exposed (E), asymptomatic (A), infectious (I), recovered (R), dead (D) | [[13-22](#_ENREF_13)] |
| **SECI_N_I_S_RD** | susceptible (S), exposed (E), mild  or no symptoms (C), non-severe (I_N_), severe (I_S_), recovered (R), dead (D) | [[23](#_ENREF_23)] |
| **SEI_p_I_c_I_s_R** | susceptible (S), exposed (E), preclinical (I_p_), clinical (I_c_), subclinical (I_s_)^2^, recovered (R) | [[24](#_ENREF_24)] |
| **SEAI_1_I_2_R**  **SEI_1_I_2_RD** | susceptible (S), exposed (E), asymptomatic (A), non-critical (I_1_), critical (I_2_), recovered (R), dead (D) | [[25](#_ENREF_25), [26](#_ENREF_26)] |
| **SEIMCRD** | susceptible (S), exposed (E), symptomatic (I), mild (M), critical (C), recovered (R), dead (D) | [[27](#_ENREF_27)] |
| **SEAIRB** | susceptible (S), exposed (E), asymptomatic (A), symptomatic (I), recovered (R), The concentration of the corona virus in the environment (B) | [[28-30](#_ENREF_28)] |
| **SLIADRE** | susceptible (S), latent (L), highly symptomatic (I), low symptomatic (A), detected (D), removed/recovered (R), environment containing viruses (E) | [[31](#_ENREF_31)] |

^1^ Transitional: the intermediate compartment of exposed to infected.

^2^ Subclinical: the infected people with few or no symptoms; clinical: the infected people with obvious symptoms

**References**

1. Efimov D and Ushirobira R. On an interval prediction of COVID-19 development based on a SEIR epidemic model. Annu Rev Control. 2021;477-487.

2. Raimúndez E, Dudkin E, Vanhoefer J, Alamoudi E, Merkt S, Fuhrmann L, et al. COVID-19 outbreak in Wuhan demonstrates the limitations of publicly available case numbers for epidemiological modeling. Epidemics. 2021;34:100439.

3. Tiwari V, Deyal N and Bisht N S. Mathematical Modeling Based Study and Prediction of COVID-19 Epidemic Dissemination Under the Impact of Lockdown in India. Front Phys. 2020;8:443.

4. Maugeri A, Barchitta M, Battiato S and Agodi A. Estimation of Unreported Novel Coronavirus (SARS-CoV-2) Infections from Reported Deaths: A Susceptible-Exposed-Infectious-Recovered-Dead Model. J Clin Med. 2020;9:1350.

5. Bae T W, Kwon K K and Kim K H. Mass Infection Analysis of COVID-19 Using the SEIRD Model in Daegu- Gyeongbuk of Korea from April to May, 2020. J Korean Med Sci. 2020;35:e317.

6. Benneyan J C, Gehrke C, Ilies I and Nehls N. Potential Community and Campus Covid-19 Outcomes Under University and College Reopening Scenarios. medRxiv. 2020. doi:10.1101/2020.08.29.20184366.

7. Han C, Li M, Haihambo N, Babuna P, Liu Q, Zhao X, et al. Mechanisms of recurrent outbreak of COVID-19: a model-based study. Nonlinear Dyn. 2021;166:1169-1185.

8. Eastman B, Meaney C, Przedborski M and Kohandel M. Modeling the impact of public response on the COVID-19 pandemic in Ontario. Plos One. 2021;16:e0249456.

9. Liu F, Wang J, Liu J, Li Y, Liu D, Tong J, et al. Predicting and analyzing the COVID-19 epidemic in China: Based on SEIRD, LSTM and GWR models. Plos One. 2020;15:e0238280.

10. Kumar P, Erturk V S and Murillo-Arcila M. A new fractional mathematical modelling of COVID-19 with the availability of vaccine. Results Phys. 2021;24:104213.

11. Reiner R C, Jr., Barber R M, Collins J K, Zheng P, Adolph C, Albright J, et al. Modeling COVID-19 scenarios for the United States. Nat Med. 2021;27:94-105.

12. Asai Y, Tsuzuki S, Kutsuna S, Hayakawa K and Ohmagari N. Effect of evacuation of Japanese residents from Wuhan, China, on preventing transmission of novel coronavirus infection: A modelling study. J Infect Chemother. 2021;27:515-520.

13. Liu X X, Fong S J, Dey N, Crespo R G and Herrera-Viedma E. A new SEAIRD pandemic prediction model with clinical and epidemiological data analysis on COVID-19 outbreak. Appl Intell. 2021;51:4162-4198.

14. Zhao Q, Wang Y, Yang M, Li M, Zhao Z, Lu X, et al. Evaluating the effectiveness of measures to control the novel coronavirus disease 2019 in Jilin Province, China. BMC Infect Dis. 2021;21:245.

15. Basnarkov L. SEAIR Epidemic spreading model of COVID-19. Chaos Solitons Fractals. 2021;142:110394.

16. Avila-Ponce de León U, Pérez Á G C and Avila-Vales E. An SEIARD epidemic model for COVID-19 in Mexico: Mathematical analysis and state-level forecast. Chaos Solitons Fractals. 2020;140:110165.

17. Li Y, Hou S, Zhang Y, Liu J, Fan H and Cao C. Effect of Travel Restrictions of Wuhan City Against COVID-19: A Modified SEIR Model Analysis. Disaster Med Public Health Prep. 2021;1-7.

18. Li W, Fu X, Sun Y and Liu M. Dynamical Analysis of a Mathematical Model of COVID-19 Spreading on Networks. Front Phys. 2021;8:607.

19. Jia L and Chen W. Uncertain SEIAR model for COVID-19 cases in China. FUZZY OPTIM DECIS MA. 2021;20:243-259.

20. Chen T M, Rui J, Wang Q P, Zhao Z Y, Cui J A and Yin L. A mathematical model for simulating the phase-based transmissibility of a novel coronavirus. Infect Dis Poverty. 2020;9:24.

21. Chen M, Li M, Hao Y, Liu Z, Hu L and Wang L. The introduction of population migration to SEIAR for COVID-19 epidemic modeling with an efficient intervention strategy. Inf Fusion. 2020;64:252-258.

22. de la Sen M, Ibeas A and Agarwal R P. On Confinement and Quarantine Concerns on an SEIAR Epidemic Model with Simulated Parameterizations for the COVID-19 Pandemic. Symmetry-Basel. 2020;12:1646.

23. Frost I, Craig J, Osena G, Hauck S, Kalanxhi E, Schueller E, et al. Modelling COVID-19 transmission in Africa: countrywise projections of total and severe infections under different lockdown scenarios. Bmj Open. 2021;11:e044149.

24. Davies N G, Kucharski A J, Eggo R M, Gimma A and Edmunds W J. Effects of non-pharmaceutical interventions on COVID-19 cases, deaths, and demand for hospital services in the UK: a modelling study. Lancet Public Health. 2020;5:e375-e385.

25. Wickramaarachchi W, Perera S S N and Jayasinghe S. COVID-19 Epidemic in Sri Lanka: A Mathematical and Computational Modelling Approach to Control. Comput Math Methods Med. 2020;2020:4045064.

26. Batabyal S and Batabyal A. Mathematical computations on epidemiology: a case study of the novel coronavirus (SARS‑CoV‑2). Theory Biosci. 2021;14:123-138.

27. Yang P, Qi J, Zhang S, Wang X, Bi G, Yang Y, et al. Feasibility study of mitigation and suppression strategies for controlling COVID-19 outbreaks in London and Wuhan. Plos One. 2020;15:e0236857.

28. Alqarni M S, Alghamdi M, Muhammad T, Alshomrani A S and Khan M A. Mathematical modeling for novel coronavirus (COVID-19) and control. Numer Methods Partial Differ Equ. 2020;38:760-776.

29. Alzahrani E, El-Dessoky M M and Baleanu D. Mathematical modeling and analysis of the novel Coronavirus using Atangana–Baleanu derivative. Results Phys. 2021;25:104240.

30. Mwalili S, Kimathi M, Ojiambo V, Gathungu D and Mbogo R. SEIR model for COVID-19 dynamics incorporating the environment and social distancing. BMC Res Notes. 2020;13:352.

31. Danchin A, Ng T W and Turinici G. A New Transmission Route for the Propagation of the SARS-CoV-2 Coronavirus. Biology (Basel). 2020;10:10.
