# Supplementary material for: Compartmental structures used in modeling COVID-19: a scoping review
Source: Infect Dis Poverty. 2022 Jun 21;11:72. doi: 10.1186/s40249-022-01001-y (PMC9209832; doi:10.1186/s40249-022-01001-y)
Supplement: Supplementary file 2 — Additional file 2. Expanded compartmental structures based on SEIR according to public health interventions. [file 40249_2022_1001_MOESM2_ESM.docx]

Table Expanded compartmental structures based on SEIR according to public health interventions.

| **Model structure** | **Interpretation** | **Reference** |
| --- | --- | --- |
| **SPEIR** | susceptible (S), protected susceptible^1^ (P), exposed (E), infectious (I), recovered (R) | [[1](#_ENREF_1)] |
| **SCEIQR** | susceptible (S), confined susceptible^1^ (C), exposed (E), infectious (I), quarantined (Q), removed (R) | [[2](#_ENREF_2)] |
| **SS_f_EIQR** | susceptible (S), behavior-changed susceptible^1^ (S_f_), exposed (E), infectious (I), hospital-quarantined (Q), recovered (R) | [[3](#_ENREF_3), [4](#_ENREF_4)] |
| **SMUEIHR** | susceptible (S), masked (M), unmasked (U), exposed (E), infectious (I), hospitalized (H), removed (R) | [[5](#_ENREF_5)] |
| **SEIHR** | susceptible (S), exposed (E), infectious (I), hospitalized/quarantined (H), removed (R) | [[6-12](#_ENREF_6)] |
| **SEIQR,SEIQRW** | susceptible (S), exposed (E), infectious (I), quarantined (Q), removed (R), the density of pathogens in the environment (W) | [[13-22](#_ENREF_13)] |
| **SEIQHR** | susceptible (S), exposed (E), infectious (I), self-isolation/quarantined (Q), isolated infectious /hospitalized (H), recovered (R) | [[23-31](#_ENREF_23)] |
| **SEIS_q_E_q_HRD** | susceptible (S), exposed (E), infectious (I), quarantined susceptible (S_q_), isolated exposed (E_q_), isolated infected (H), recovered (R), dead (D) | [[32-35](#_ENREF_32)] |
| **SEMIR** | susceptible (S), exposed (E), missed cases (M), infectious (I), recovered (R) | [[36](#_ENREF_36)] |
| **SEICR,SEICQR** | susceptible (S), exposed (E), infectious (I), confirmed (C), quarantined(Q), recovered (R) | [[37](#_ENREF_37), [38](#_ENREF_38)] |
| **SEQ_1_IQ_2_R** | susceptible (S), exposed (E), suspected population under home quarantine (Q_1_), infectious (I), medical quarantine population of confirmed cases (Q_2_), recovered (R) | [[39](#_ENREF_39)] |
| **SEI_u_I_r_QR** | susceptible (S), exposed (E), Infectious individuals that be tested/ reported/ diagnosed (I_r_), infectious individuals that not be tested/ reported/ diagnosed (I_u_), quarantined/hospitalized (Q), removed (R) | [[40-42](#_ENREF_40)] |
| **SS_q_EE_q_PIQR** | susceptible (S), quarantined susceptible (S_q_), exposed (latent) (E), isolated exposed (E_q_), isolated suspected (P), undiagnosed and non-isolated infectious (I), confirmed and isolated infectious (Q), recovered (R) | [[43](#_ENREF_43)] |
| **SS_q_EI_1_I_2_HVR** | susceptible (S), self-quarantine susceptible (S_q_), exposed (E), infectious people with timely diagnosis (I_1_), infectious people with delayed diagnosis (I_2_), hospitalized (H), the virus in the environment(V), recovered (R) | [[44](#_ENREF_44)] |

^1．^Protected, confined and behavior changed susceptible people are less likely to be infected than ordinary susceptible people.

**References**

1. Al-Khani A M, Khalifa M A, Almazrou A and Saquib N. The SARS-CoV-2 pandemic course in Saudi Arabia: A dynamic epidemiological model. Infect Dis Model. 2020;5:766-771.

2. Grzybowski J M V, da Silva R V and Rafikov M. Expanded SEIRCQ Model Applied to COVID-19 Epidemic Control Strategy Design and Medical Infrastructure Planning. MATH PROBL ENG. 2020;2020:8198563.

3. Kim S, Seo Y B and Jung E. Prediction of COVID-19 transmission dynamics using a mathematical model considering behavior changes in Korea. Epidemiol Health. 2020;42:e2020026.

4. Kim S, Ko Y, Kim Y J and Jung E. The impact of social distancing and public behavior changes on COVID-19 transmission dynamics in the Republic of Korea. Plos One. 2020;15:e0238684.

5. Mumbu A J and Hugo A K. Mathematical modelling on COVID-19 transmission impacts with preventive measures: a case study of Tanzania. J Biol Dyn. 2020;14:748-766.

6. Asamoah J K K, Bornaa C S, Seidu B and Jin Z. Mathematical analysis of the effects of controls on transmission dynamics of SARS-CoV-2. ALEX ENG J. 2020;59:5069-5078.

7. Xing G R, Li M T, Li L and Sun G Q. The Impact of Population Migration on the Spread of COVID-19: A Case Study of Guangdong Province and Hunan Province in China. Front Phys. 2020;8:488.

8. Kim S, Jeong Y D, Byun J H, Cho G, Park A, Jung J H, et al. Evaluation of COVID-19 epidemic outbreak caused by temporal contact-increase in South Korea. Int J Infect Dis. 2020;96:454-457.

9. Yang C, Yang Y, Li Z and Zhang L. Modeling and analysis of COVID-19 based on a time delay dynamic model. Math Biosci Eng. 2020;18:154-165.

10. He S, Peng Y and Sun K. SEIR modeling of the COVID-19 and its dynamics. Nonlinear Dyn. 2020;101:1667-1680.

11. Hussain T, Ozair M, Ali F, Rehman S U, Assiri T A and Mahmoud E E. Sensitivity analysis and optimal control of COVID-19 dynamics based on SEIQR model. Results Phys. 2021;22:103956.

12. Peter O J, Qureshi S, Yusuf A, Al-Shomrani M and Idowu A A. A new mathematical model of COVID-19 using real data from Pakistan. Results Phys. 2021;24:104098.

13. Zhang Z, Zeb A, Egbelowo O F and Erturk V S. Dynamics of a fractional order mathematical model for COVID-19 epidemic. Adv Differ Equ. 2020;2020:420.

14. Mandal M, Jana S, Nandi S K, Khatua A, Adak S and Kar T K. A model based study on the dynamics of COVID-19: Prediction and control. Chaos Solitons Fractals. 2020;136:109889.

15. Chanu A L and Singh R K B. Stochastic approach to study control strategies of Covid-19 pandemic in India. Epidemiol Infect. 2020;148:e200.

16. Youssef H, Alghamdi N, Ezzat M A, El-Bary A A and Shawky A M. Study on the SEIQR model and applying the epidemiological rates of COVID-19 epidemic spread in Saudi Arabia. Infect Dis Model. 2021;6:678-692.

17. Ryu S, Ali S T, Lim J S and Chun B C. Estimation of the Excess COVID-19 Cases in Seoul, South Korea by the Students Arriving from China. Int J Environ Res Public Health. 2020;17:3113.

18. Adhikari K, Gautam R, Pokharel A, Uprety K N and Vaidya N K. Transmission dynamics of COVID-19 in Nepal: Mathematical model uncovering effective controls. J Theor Biol. 2021;521:110680.

19. Collins O C and Duffy K J. Estimating the impact of lock-down, quarantine and sensitization in a COVID-19 outbreak: lessons from the COVID-19 outbreak in ChinaE. Peerj. 2020;8:e9933.

20. Zhang B, Zhou H and Zhou F. Study on SARS-CoV-2 transmission and the effects of control measures in China. Plos One. 2020;15:e0242649.

21. Raza A, Ahmadian A, Rafiq M, Salahshour S and Ferrara M. An analysis of a nonlinear susceptible-exposed-infected-quarantine-recovered pandemic model of a novel coronavirus with delay effect. Results Phys. 2021;21:103771.

22. Kim B N, Kim E, Lee S and Oh C. Mathematical Model of COVID-19 Transmission Dynamics in South Korea: The Impacts of Travel Restrictions, Social Distancing, and Early Detection. Processes. 2020;8:1304.

23. Raslan W E. Fractional mathematical modeling for epidemic prediction of COVID-19 in Egypt. Ain Shams Eng J. 2021;12:3057-3062.

24. Memon Z, Qureshi S and Memon B R. Assessing the role of quarantine and isolation as control strategies for COVID-19 outbreak: A case study. Chaos Solitons Fractals. 2021;144:110655.

25. Madubueze C E, Dachollom S and Onwubuya I O. Controlling the Spread of COVID-19: Optimal Control Analysis. Comput Math Methods Med. 2020;2020:6862516.

26. Dwomoh D, Iddi S, Adu B, Aheto J M, Sedzro K M, Fobil J, et al. Mathematical modeling of COVID-19 infection dynamics in Ghana: Impact evaluation of integrated government and individual level interventions. Infect Dis Model. 2021;6:381-397.

27. Shah N H, Sheoran N, Jayswal E, Shukla D, Shukla N, Shukla J, et al. Modelling COVID-19 transmission in the United States through interstate and foreign travels and evaluating impact of governmental public health interventions. J Math Anal Appl. 2020;2020:124896.

28. Balike Dieudonné Z. Mathematical model for the mitigation of the economic effects of the Covid-19 in the Democratic Republic of the Congo. Plos One. 2021;16:e0250775.

29. Javeed S, Anjum S, Alimgeer K S, Atif M, Khan M S, Farooq W A, et al. A Novel Mathematical Model for COVID-19 with Remedial Strategies. Results Phys. 2021;27:104248.

30. Lopman B, Liu C Y, Le Guillou A, Handel A, Lash T L, Isakov A P, et al. A modeling study to inform screening and testing interventions for the control of SARS-CoV-2 on university campuses. Sci Rep. 2021;11:5900.

31. Prathumwan D, Trachoo K and Chaiya I. Mathematical Modeling for Prediction Dynamics of the Coronavirus Disease 2019 (COVID-19) Pandemic, Quarantine Control Measures. Symmetry-Basel. 2020;12:1404.

32. Su L, Hong N, Zhou X, He J, Ma Y, Jiang H, et al. Evaluation of the Secondary Transmission Pattern and Epidemic Prediction of COVID-19 in the Four Metropolitan Areas of China. Front Med (Lausanne). 2020;7:171.

33. Ding Y and Gao L. An evaluation of COVID-19 in Italy: A data-driven modeling analysis. Infect Dis Model. 2020;5:495-501.

34. Hu Z, Cui Q, Han J, Wang X, Sha W E I and Teng Z. Evaluation and prediction of the COVID-19 variations at different input population and quarantine strategies, a case study in Guangdong province, China. Int J Infect Dis. 2020;95:231-240.

35. Cui Q, Hu Z, Li Y, Han J, Teng Z and Qian J. Dynamic variations of the COVID-19 disease at different quarantine strategies in Wuhan and mainland China. J Infect Public Health. 2020;13:849-855.

36. Hu B, Dehmer M, Emmert-Streib F and Zhang B. Analysis of the real number of infected people by COVID-19: A system dynamics approach. Plos One. 2021;16:e0245728.

37. Huang B, Zhu Y, Gao Y, Zeng G, Zhang J, Liu J, et al. The analysis of isolation measures for epidemic control of COVID-19. Appl Intell (Dordr). 2021;51:3074-3085.

38. Sun G-Q, Wang S-F, Li M-T, Li L, Zhang J, Zhang W, et al. Transmission dynamics of COVID-19 in Wuhan, China: effects of lockdown and medical resources. Nonlinear Dynamics. 2020;101:1981-1993.

39. Mishra B K, Keshri A K, Saini D K, Ayesha S, Mishra B K and Rao Y S. Mathematical model, forecast and analysis on the spread of COVID-19. Chaos Solitons Fractals. 2021;147:110995.

40. Post R A J, Regis M, Zhan Z and van den Heuvel E R. How did governmental interventions affect the spread of COVID-19 in European countries? Bmc Public Health. 2021;21:411.

41. Capuano F. Modeling Growth, Containment and Decay of the COVID-19 Epidemic in Italy. Front Phys. 2020;8:554.

42. Giamberardino P D, Iacoviello D, Papa F and Sinisgalli C. Dynamical Evolution of COVID-19 in Italy With an Evaluation of the Size of the Asymptomatic Infective Population. IEEE J Biomed Health Inform. 2021;25:1326-1332.

43. Zu J, Li M L, Li Z F, Shen M W, Xiao Y N and Ji F P. Transmission patterns of COVID-19 in the mainland of China and the efficacy of different control strategies: a data- and model-driven study. Infect Dis Poverty. 2020;9:83.

44. Huang J and Qi G. Effects of control measures on the dynamics of COVID-19 and double-peak behavior in Spain. Nonlinear Dyn. 2020;101:1-11.
