# Supplementary material for: Compartmental structures used in modeling COVID-19: a scoping review
Source: Infect Dis Poverty. 2022 Jun 21;11:72. doi: 10.1186/s40249-022-01001-y (PMC9209832; doi:10.1186/s40249-022-01001-y)
Supplement: Supplementary file 3 — Additional file 3. Compartmental structures of compartmental models for COVID-19 considered age structures. [file 40249_2022_1001_MOESM3_ESM.docx]

Table Structures of compartmental models for COVID-19 considered age structures^1^.

| **Age structure** | **Compartmental structure** | **Interpretation** | **Reference** |
| --- | --- | --- | --- |
| **0**−**9, 10**−**19, …, 70**−**79, 80+** | **S_i_E_i_I_i_R_i_D_i_** | susceptible (S_i_), exposed (E_i_), infected (I_i_), recovered (R_i_), dead (D_i_) in age group i | [[1](#_ENREF_1)] |
| **0**−**9, 10**−**19, …, 60**−**69, 70+** | **S_i_E_i_I_pi_I_ci_I_si_R_i_** | susceptible (S_i_), exposed (E_i_), pre-symptomatic (I_pi_), clinically (I_ci_), subclinical (I_si_), recovered (R_i_) in age group i | [[2](#_ENREF_2)] |
| **0**−**14, 15**−**44, 45**−**64, 65+^1^**  **0**−**4, 5**−**9, 10**−**14…**  **0**−**18, 19**−**30, 31**−**70, 70+** | **S_i_E_i_A_i_I_i_R_i_, S_i_E_i_P_i_A_i_I_i_R_i_ ,**  **S_i_E_i_P_i_A_i_I_i_R_i_D_i_** | susceptible (S_i_), exposed (E_i_), pre-symptomatic (P_i_), asymptomatic (A_i_), infected (I_i_), recovered (R_i_) ,dead (D_i_) in age group i | [[3-5](#_ENREF_3)] |
| **0**−**4, 5**−**9, 10**−**14, …, 75**−**70, 80+** | **S_i_E_i_I_i_I_si_R_Ai_R_Ui_R_Hi_D_i_** | susceptible (S_i_), exposed (E_i_), infected (I_i_), asymptomatic recovered individuals (R_Ai_), mild recovered (R_Mi_), severe (I_si_), severe recovered (R_Hi_), dead (D_i_) in age group i | [[6](#_ENREF_6)] |
| **0**−**19, 20**−**59, 60+ 0**−**19, 20**−**29, …, 70**−**79, 80+**  **0**−**9, 10**−**19, …, 60**−**69, 70+** | **S_i_E_i_I_i_Q_i_R_i_** | susceptible (S_i_), exposed (E_i_), infected (I_i_), hospitalized / quarantined (Q_i_), recovered (R_i_) in age group i | [[7-9](#_ENREF_7)] |
| **0**−**15, 15**−**29, 30**−**59, 59+**  **0**−**17, 18**−**44, 45**−**64, 65**−**74, 75+** | **S_i_E_i_A_i_M_i_H_i_C_i_R_i_,**  **S_i_E_i_M_i_H_i_C_i_R_i_D_i_** | susceptible (S_i_), exposed (E_i_), asymptomatic (A_i_), mild (M_i_), severe (H_i_), critical (C_i_), recovered (R_i_), dead (D_i_) in age group i | [[10](#_ENREF_10), [11](#_ENREF_11)] |
| **0**−**9, 10**−**19, …, 70**−**79, 80+** | **S_i_E_i_I_i_Q_Si_Q_Ei_Q_Ii_R_i_** | susceptible (S_i_), exposed (E_i_), quarantined susceptible (Q_Si_), quarantined exposed (Q_Ei_), infected (I_i_), isolated infected (Q_Ii_), recovered (R_i_) in age group i | [[12](#_ENREF_12)] |
| **0**−**14, 15**−**49, 50**−**69, 70**−**80, 80+** | **S_i_E_i_L_i_I_i_R_i_T_pi_A_si_S_si_S_vi_C_ri_R_di_D_i_** | susceptible (S_i_), exposed (E_i_), post latency (L_i_), infectious (I_i_), undocumented recovered (R_i_), tested positive (T_pi_), asymptomatic (A_si_), symptomatic (S_si_), severe (S_vi_), critical (C_ri_), dead (D_i_), documented recovered (R_di_) in age group i | [[13](#_ENREF_13)] |
| **0**−**18, 19**−**64, 65+** | **S_i_E_i_I_ai_I_mi_I_hi_Q_i_R_i_D_i_** | susceptible (S_i_), exposed (E_i_), asymptomatic (I_ai_), mildly infected and symptomatic (I_mi_), severely infected, symptomatic, and hospitalized (I_hi_), detected infections and isolated at home (Q_i_), recovered/removed (R_i_), dead (D_i_) in age group i | [[14](#_ENREF_14)] |
| **0**−**4, 5**−**9, 10**−**14, …, 75**−**70, 80+** | **S_i_E_i_A_i_B_i_C_i_H_i_Q_i_R_i_D_i_** | susceptible (S_i_), exposed (E_i_), asymptomatic (A_i_), symptomatic before (B_i_), self-isolation (C_i_), hospitalized in MCU (H_i_)^1^, hospitalized in ICU (Q_i_), removed (R_i_), deceased (D_i_) in age group i | [[15](#_ENREF_15)] |
| **0–4, 5–14, 15–29, 30–59, 60–69, 70–79, 80+** | **S_i_E_i_I_pi_I_ai_I_si_I_ci_I_hi_R_i_D_i_** | susceptible (S_i_), latent (E_i_), pre-symptomatic (I_pi_), asymptomatic infectious (I_ai_), symptomatic infectious (I_si_), hospital care (I_hi_), critical care (I_ci_), recovered (R_i_), dead (D_i_) in age group i | [[16](#_ENREF_16)] |
| **0**−**14, 15**−**49, 50**−**69, 70+** | **S_ij_E_ij_I_ij_Q_ij_H_ij_R_ij_D_ij_** | susceptible (S_ij_), exposed (E_ij_), pre-symptomatic (Ip_ij_), mild to moderate (I_mij_), severe (I_sij_), quarantined & exposed (Q_Eij_), pre-symptomatic & isolated (Q_Ipij_), mild to moderate &isolated (Q_Imij_), severe &isolated (Q_Isij_), Isolated (Q_ij_), admitted to hospital (H_ij_), pre-ICU (P_ICUij_), ICU (H_ICUij_), recovered (R_ij_), dead (D_ij_) in age group i and health statu j | [[17](#_ENREF_17)] |
| **0**−**14, 15**−**24, 25**−**54, 55**−**64, 65+** | **S_i_E_i_I_i_Q_i_H_i_R_i_D_i_** | susceptible (S_i_), exposed (E_i_), self-quarantine before infectiousness (Q_si_), sub-clinical infectious that have no symptoms or only mild symptoms (I_sci_), asymptomatic individuals that be isolated in home (Q_sci_), clinical infectious that have severe symptoms (I_ci_), hospitalized (H_i_), critical care in an ICU (I_icui_), recovered (R_i_), dead (D_i_) in age group i | [[18](#_ENREF_18)] |
| **0**−**10, 10**−**20, …,70**−**80, 80+** | **S_i_C_i_E_i_A_i_I_i_Q_i_H_i_R_i_** | susceptible (S_i_), subset of susceptible removed from the epidemics (C_i_), exposed (E_i_), asymptomatic (A_i_), symptomatic (I_i_), quarantined (Q_i_), hospitalized (H_i_), recovered (R_i_) in age group i | [[19](#_ENREF_19)] |
| **0**−**9, 10**−**19, …, 70**−**79, 80+** | **S_i_E_i_A_i_I_i_H_i_R_i_D_i_** | susceptible (S_i_), latently infected (E_i_), asymptomatic infectious (A_i_), infectious individuals with symptoms/clinically ill (I_i_), hospitalized patients (H_i_), recovered (R_i_), died due to disease (D_i_) in group i | [[20](#_ENREF_20)] |
| **0**−**10, 10**−**20, …, 60**−**70, 70+** | **S_i_V_i_E_i_E_vi_A_i_A_vi_I_i_Q_i_R_i_R_vi_D_i_** | susceptible (S_i_), vaccinated (V_i_), exposed (Ei), exposed& vaccinated (E_Vi_), asymptomatic (A_i_), asymptomatic & vaccinated (A_Vi_), symptomatic (I_i_), isolated (Q_i_), recovered (R_i_), recovered & vaccinated (R_Vi_), died (D_i_) in age group i | [[21](#_ENREF_21)] |
|  |  |  |  |

^1^ Different age structures but with same or similar compartment structures in different studies.

^2^ MCU: middle care unit.

**References**

1. Cintra H P C and Fontinele F N. Estimative of real number of infections by COVID-19 in Brazil and possible scenarios. Infect Dis Model. 2020;5:720-736.

2. Davies N G, Klepac P, Liu Y, Prem K, Jit M and Eggo R M. Age-dependent effects in the transmission and control of COVID-19 epidemics. Nat Med. 2020;26:1205-1211.

3. Zhao Z Y, Zhu Y Z, Xu J W, Hu S X, Hu Q Q, Lei Z, et al. A five-compartment model of age-specific transmissibility of SARS-CoV-2. Infect Dis Poverty. 2020;9:117.

4. Jentsch P C, Anand M and Bauch C T. Prioritising COVID-19 vaccination in changing social and epidemiological landscapes: a mathematical modelling study. Lancet Infect Dis. 2021;21:1097-1106.

5. Rǎdulescu A, Williams C and Cavanagh K. Management strategies in a SEIR-type model of COVID 19 community spread. Sci Rep. 2020;10:21256.

6. Kyrychko Y N, Blyuss K B and Brovchenko I. Mathematical modelling of the dynamics and containment of COVID-19 in Ukraine. Sci Rep. 2020;10:19662.

7. Gondim J A M and Machado L. Optimal quarantine strategies for the COVID-19 pandemic in a population with a discrete age structure. Chaos Solitons Fractals. 2020;140:110166.

8. Choi Y, Kim J S, Choi H, Lee H and Lee C H. Assessment of Social Distancing for Controlling COVID-19 in Korea: An Age-Structured Modeling Approach. Int J Environ Res Public Health. 2020;17:7474.

9. Rozhnova G, van Dorp C H, Bruijning-Verhagen P, Bootsma M C J, van de Wijgert J, Bonten M J M, et al. Model-based evaluation of school- and non-school-related measures to control the COVID-19 pandemic. Nat Commun. 2021;12:1614.

10. Kimathi M, Mwalili S, Ojiambo V and Gathungu D K. Age-structured model for COVID-19: Effectiveness of social distancing and contact reduction in Kenya. Infect Dis Model. 2021;6:15-23.

11. Albani V V L, Velho R M and Zubelli J P. Estimating, monitoring, and forecasting COVID-19 epidemics: a spatiotemporal approach applied to NYC data. Sci Rep. 2021;11:9089.

12. Lee T, Kwon H D and Lee J. The effect of control measures on COVID-19 transmission in South Korea. Plos One. 2021;16:e0249262.

13. Verma V R, Saini A, Gandhi S, Dash U and Koya S F. Capacity-need gap in hospital resources for varying mitigation and containment strategies in India in the face of COVID-19 pandemic. Infect Dis Model. 2020;5:608-621.

14. Vatcheva K P, Sifuentes J, Oraby T, Maldonado J C, Huber T and Villalobos M C. Social distancing and testing as optimal strategies against the spread of COVID-19 in the Rio Grande Valley of Texas. Infect Dis Model. 2021;6:729-742.

15. Balabdaoui F and Mohr D. Age-stratified discrete compartment model of the COVID-19 epidemic with application to Switzerland. Sci Rep. 2020;10:21306.

16. Röst G, Bartha F A, Bogya N, Boldog P, Dénes A, Ferenci T, et al. Early Phase of the COVID-19 Outbreak in Hungary and Post-Lockdown Scenarios. Viruses. 2020;12:708.

17. Tuite A R, Fisman D N and Greer A L. Mathematical modelling of COVID-19 transmission and mitigation strategies in the population of Ontario, Canada. Cmaj. 2020;192:E497-E505.

18. Chadsuthi S and Modchang C. Modelling the effectiveness of intervention strategies to control COVID-19 outbreaks and estimating healthcare demand in Germany. Public Health Pract (Oxf). 2021;2:100121.

19. Lyra W, do Nascimento J D, Jr., Belkhiria J, de Almeida L, Chrispim P P M and de Andrade I. COVID-19 pandemics modeling with modified determinist SEIR, social distancing, and age stratification. The effect of vertical confinement and release in Brazil. Plos One. 2020;15:e0237627.

20. Stanojevic S, Ponjavic M, Stanojevic S, Stevanovic A and Radojicic S. Simulation and prediction of spread of COVID-19 in The Republic of Serbia by SEIRDS model of disease transmission. Microb Risk Anal. 2021;100161.

21. Foy B H, Wahl B, Mehta K, Shet A, Menon G I and Britto C. Comparing COVID-19 vaccine allocation strategies in India: A mathematical modelling study. Int J Infect Dis. 2021;103:431-438.
