# Supplementary material for: Compartmental structures used in modeling COVID-19: a scoping review
Source: Infect Dis Poverty. 2022 Jun 21;11:72. doi: 10.1186/s40249-022-01001-y (PMC9209832; doi:10.1186/s40249-022-01001-y)
Supplement: Supplementary file 4 — Additional file 4. Compartmental structures that considered both virus characteristics and interventions. [file 40249_2022_1001_MOESM4_ESM.docx]

Table Compartmental structures that considered both virus characteristics and interventions.

| **Model structure** | **Interpretation** | **Reference** |
| --- | --- | --- |
| **SUEIQRD, SUEIAQRD, SUEIHCDR** | susceptible (S), unsusceptible^1^ (U), exposed (E), asymptomatic (A), infected (symptomatic) (I), quarantined (Q), hospitalized (H), critical (C), recovered (R), dead (D) | [[1-4](#_ENREF_1)] |
| **SEAIE_q_A_q_I_q_R_1_R_2_** | susceptible (S), exposed (E), asymptomatic (A), infected (symptomatic) (I), quarantined exposed (E_q_), detected asymptomatic carriers and detected mild carries (A_q_), quarantined infectious individuals with obvious symptom (I_q_), quarantined removed (R_1_), removed without quarantine (R_2_) | [[5](#_ENREF_5)] |
| **SLEAICR** | susceptible (S), lockdown^1^ (L), exposed (E), asymptomatic (A), symptomatic (I), hospitalized (C), recovered (R) | [[6](#_ENREF_6)] |
| **SEIAS_q_E_q_R** | susceptible (S), exposed (E), mild (I_1_), ordinary (I_2_), severe (I_3_), critical (I_4_), asymptomatic (A), quarantined susceptible (S_q_), quarantined exposed (E_q_), removed (R) | [[7](#_ENREF_7)] |
| **SEIAS_i_QHR_h_RD** | susceptible (S), exposed (E), symptomatic(I), asymptomatic (A), isolated susceptible (S_i_), quarantined infected (Q), hospitalized (H), recovered population from hospitals (R_h_), Recovered (R), dead (D) | [[8](#_ENREF_8)] |
| **SEI_1_I_2_R** | susceptible (S), exposed (E), infected (I_1_), infected after receiving ineffective treatment (I_2_), recovered (R) | [[9](#_ENREF_9)] |
| **SQ_S_S_T_EAIQHGR** | susceptible (S), isolated susceptible (Q_s_), susceptible persons removed from isolation^2^ (S_T_), exposed (E), asymptomatic (A), symptomatic (I), isolated infected (Q), hospitalized (H), severe disease and are admitted to ICUs (G), recovered (R) | [[10](#_ENREF_10)] |
| **SEIWRCD** | susceptible (S), exposed (E), infected (I), recovered(R3), hospitalized that never require an intensive care bed (W_1_), recovered from non-ICU (R1), deaths from non-ICU (D1), hospitalized that require an intensive care bed (W_2_), ICU patients (C), recovered from ICU(R_2_), deaths from ICU(D_2_) | [[11](#_ENREF_11)] |
| **SEAII_A_R_A_MHH_ICU_R_S_D** | susceptible (S), exposed (E), asymptomatic (A), symptomatic (I), Isolated asymptomatic (I_A_), recovered from asymptomatic cases (R_A_), mild (M), hospitalized (H), in critical care (H_ICU_), recovered from symptomatic cases (R_S_), deceased (D) | [[12](#_ENREF_12)] |
| **SEAIQ_S_Q_E_Q_I_Q_A_R** | susceptible (S), exposed (E), infected (I), asymptomatic (A), self-quarantined (Q_S_), quarantine of exposed (Q_E_), isolated (Q_I_), quarantine of asymptomatic (Q_A_), recovered(R) | [[13](#_ENREF_13)] |
| **SEE_q_I_s_I_a_H_1_H_2_RD** | susceptible (S), exposed (E), exposed and quarantined (Eq), symptoms (Is), asymptomatic (Ia), confirmed cases quarantined at home (H_1_), confirmed cases quarantined at home (H_2_), recovered (R), dead(D) | [[14](#_ENREF_14)] |
| **SEI_p_I_c_HH_ICU_RX** | susceptible (S), exposed (E), chronically infected (I_c_), primarily infected (I_p_), hospitalized (H), patients in ICUs (H_ICU_), recovered (immune) (R), deceased (X) | [[15](#_ENREF_15)] |
| **SEIR_Is_R_Ds_S_q_E_q_I_q_HR_s_D_s_** | susceptible (S), exposed (E), infected (I), recovered not go to hospital (R_Is_), dead not go to hospital (R_Ds_), quarantined susceptible (S_q_), quarantined exposed (E_q_), quarantined infectious (I_q_), hospitalized (H), recovered from hospital (R_s_), dead from hospital (D_s_) | [[16](#_ENREF_16)] |
| **SEAP_s_RQ_S_Q_E_Q_A_Q_P_S_ym_** | susceptible (S), exposed (E), asymptomatic (A), pre-symptomatic infection (P_s_), recovered(R), quarantine susceptible (Q_S_), quarantine exposed (Q_E_), quarantine asymptomatic (Q_A_), quarantine pre-symptomatic (Q_P_), symptomatic (S_ym_) | [[17](#_ENREF_17)] |
| **SEI_a_I_s_Q_a_Q_s_R_u_R_a_R_s_D** | susceptible (S), infected but not yet infectious (E), undetected asymptomatic (I_a_), undetected symptomatic (I_s_), detected and quarantined asymptomatic (Q_a_), detected and quarantined symptomatic (Q_s_), undetected recovered asymptomatic (R_u_), recovered detected asymptomatic (R_a_), recovered detected symptomatic (R_s_), dead (D) | [[18](#_ENREF_18)] |
| **SEIA_u_A_d_R** | susceptible (S), exposed (E), infected (I), asymptomatic undetected (A_u_), Asymptomatic detected (A_d_), recovered (R) | [[19](#_ENREF_19)] |
| **SEAII_D_R** | susceptible (S), exposed (E), symptomatic (I), asymptomatic (A), detected infectious humans via testing (I_D_), recovered(R) | [[20](#_ENREF_20)] |
| **SEUIHRR_U_D** | susceptible (S), exposed (E), asymptomatic (U), symptomatic (I), diagnosed (H), recovered from detected (R), recovered from undetected (R_U_), deceased (D) | [[21](#_ENREF_21)] |
| **SEAITRD** | susceptible (S), exposed (E), asymptomatic (A), undiagnosed infections with mild/moderate (I_1_), undiagnosed infections with severe/critical symptoms (I_2_), diagnosed infections with mild/ moderate (T_1_), diagnosed infections with severe/critical symptoms (T_2_), recovered (R), deceased (D) | [[22](#_ENREF_22)] |
| **SEIQD_u_R_u_R_q_D_q_** | susceptible (S), exposed (E), infective(I), quarantined (Q), recovered quarantined (R_q_), dead quarantined (D_q_), undetected recovered (R_u_), undetected dead (D_u_) | [[23](#_ENREF_23)] |
| **SEI_1_I_u_HRD** | susceptible (S), exposed (E), undetected infected (I_1_), mild or asymptomatic that undetected and will remain undetected (I_u_), hospitalized/reported (H), recovered (R), dead (D) | [[24](#_ENREF_24)] |
| **SS_p_EAQI_s_HRR_A_D** | susceptible (S), susceptible individuals who partially or obey the government policy (S_p_), exposed (E), asymptomatic or mildly symptomatic(A), quarantined asymptomatic (Q), severely symptomatic (I_s_), reported hospitalized and/or isolated (H), reported recovered (R), asymptomatic individuals who recover but are not reported (R_A_), dead (D) | [[25](#_ENREF_25)] |
| **SEAI_m_I_s_QRDP** | susceptible (S), exposed(E), undetected asymptomatic (A), mild (I_m_), severe (I_s_), detected and quarantined patients (both asymptomatic and symptomatic) (Q), deceased (D), recovered (R), public’s perception (P) | [[26](#_ENREF_26)] |
| **SEPMGIR** | susceptible (S), exposed (E), pre-symptomatic individuals who are not (yet) tested (P_1_), pre-symptomatic and tested individuals who are isolated (P_2_), mildly infectious individuals who are not tested (M_1_), mildly infectious and tested individuals who are isolated (M_2_), mildly infectious individuals who are isolated (G), severely infectious individuals who are not yet hospitalized (I_1_), infectious individuals who are hospitalized due to severe symptoms (I_2_), recovered (R) | [[27](#_ENREF_27)] |
| **SEA_U_A_C_I_U_I_T_F_T_R_U_R_r_** | susceptible (S), unexposed contacts (S_C_), exposed (E), exposed contacts (E_C_), infected contacts (I_C_), untested asymptomatic (A_U_), contact traced asymptomatic (A_C_), untested infected (I_U_), tested/isolated cases (I_T_), fatalities (F_T_), untested recovered (R_U_), tested recovered (R_T_) | [[28](#_ENREF_28)] |
| **SEIHRD** | susceptible (S), exposed (E), infectious (I), infectious but undetected (I_u_), hospitalized or in quarantine at home (detected and reported) that will recover (R_H_), hospitalized that will die (H_D_), dead(D), recovered after being previously detected as infectious (R_d_), recovered after being previously infectious but undetected (R_u_) | [[29](#_ENREF_29)] |
| **SEIHQRD** | susceptible (S), exposed (E), infectious (I), infectious but undetected that will survive (I_u_), infectious but undetected that will die (ID_u_), quarantine (Q), hospitalized that will recover (H_R_), hospitalized that will die (H_D_), dead from hospital (D), dead but undetected (D_u_), recovered undetected before (R_u_), recovered after being previously detected (R_d_) | [[30](#_ENREF_30)] |
| **SVEAIQR** | susceptible (S), vaccinated (V), undetected exposed (E_u_), detected exposed (E_d_), asymptomatic (A), symptomatic (I), quarantined (Q), recovered (R) | [[31](#_ENREF_31)] |
| **SEIQRD** | susceptible (S), exposed (E), infected (I), isolation (quarantine) (Q), vaccinated and recovered (R), dead (D) | [[32](#_ENREF_32)] |
| **S_u_S_v_E_1_E_2_I_s_I_a_I_h_R** | unvaccinated susceptible (S_u_), vaccinated susceptible (S_v_), early-exposed (E_1_), pre-symptomatic (E_2_), asymptomatic (I_a_), symptomatic (I_s_), hospitalized (I_h_), recovered (R) | [[33](#_ENREF_33)] |
| **SEIQRDV, MSEIHRDV** | semi-susceptible (M), susceptible (S), exposed (E), infected (I), quarantined/hospitalized (Q/H), recovered (R), dead (D), vaccinated (V) | [[34](#_ENREF_34), [35](#_ENREF_35)] |

^1^. Unsusceptible: a susceptible person can become unsusceptible by factors such as using of facemasks, hand washing and SD (social distance).

^2^. Susceptible persons removed from isolation(S_T_): Isolated susceptible individuals, after a period, are released from isolation and transferred to compartment S_T_.

^3^. Primarily infected: individuals that stay infectious within the reported duration of the infectious period after the incubation period; chronically infected: individuals that have less infectious but stay infectious and be diagnosed for a longer duration[[15](#_ENREF_15)].

**References**

1. Kumari P, Singh H P and Singh S. SEIAQRDT model for the spread of novel coronavirus (COVID-19): A case study in India. Appl Intell (Dordr). 2021;51:2818-2837.

2. Godio A, Pace F and Vergnano A. SEIR Modeling of the Italian Epidemic of SARS-CoV-2 Using Computational Swarm Intelligence. Int J Environ Res Public Health. 2020;17:3535.

3. Pinto Neto O, Kennedy D M, Reis J C, Wang Y, Brizzi A C B, Zambrano G J, et al. Mathematical model of COVID-19 intervention scenarios for SAo Paulo-Brazil. Nat Commun. 2021;12:418.

4. Xu C, Yu Y, Chen Y and Lu Z. Forecast analysis of the epidemics trend of COVID-19 in the USA by a generalized fractional-order SEIR model. Nonlinear Dyn. 2020;101:1621-1634.

5. Wang X, Tang T, Cao L, Aihara K and Guo Q. INFERRING KEY EPIDEMIOLOGICAL PARAMETERS AND TRANSMISSION DYNAMICS OF COVID-19 BASED ON A MODIFIED SEIR MODEL. Math Model Nat Phenom. 2020;15:74.

6. Sardar T, Nadim S S, Rana S and Chattopadhyay J. Assessment of lockdown effect in some states and overall India: A predictive mathematical study on COVID-19 outbreak. Chaos Solitons Fractals. 2020;139:110078.

7. Wei Y Y, Lu Z Z, Du Z C, Zhang Z J, Zhao Y, Shen S P, et al. Fitting and forecasting the trend of COVID-19 by SEIR(+CAQ) dynamic model. Zhonghua Liu Xing Bing Xue Za Zhi. 2020;41:470-475.

8. Wan H, Cui J A and Yang G J. Risk estimation and prediction of the transmission of coronavirus disease-2019 (COVID-19) in the mainland of China excluding Hubei province. Infect Dis Poverty. 2020;9:116.

9. Oduro B and Magagula V M. COVID-19 intervention models: An initial aggressive treatment strategy for controlling the infection. Infect Dis Model. 2021;6:351-361.

10. Amaku M, Covas D T, Coutinho F A B, Azevedo R S and Massad E. Modelling the impact of contact tracing of symptomatic individuals on the COVID-19 epidemic. Clinics (Sao Paulo). 2021;76:e2639.

11. Dagpunar J S. Sensitivity of UK Covid-19 deaths to the timing of suppression measures and their relaxation. Infect Dis Model. 2020;5:525-535.

12. Mayorga L, Garcia Samartino C, Flores G, Masuelli S, Sanchez M V, Mayorga L S, et al. A modelling study highlights the power of detecting and isolating asymptomatic or very mildly affected individuals for COVID-19 epidemic management. Bmc Public Health. 2020;20:1809.

13. Ali M, Shah S T H, Imran M and Khan A. The role of asymptomatic class, quarantine and isolation in the transmission of COVID-19. J Biol Dyn. 2020;14:389-408.

14. Yu D, Zhu G, Wang X, Zhang C, Soltanalizadeh B, Wang X, et al. Assessing effects of reopening policies on COVID-19 pandemic in Texas with a data-driven transmission model. Infect Dis Model. 2021;6:461-473.

15. Tepekule B, Hauser A, Kachalov V N, Andresen S, Scheier T, Schreiber P W, et al. Assessing the potential impact of transmission during prolonged viral shedding on the effect of lockdown relaxation on COVID-19. PLoS Comput Biol. 2021;17:e1008609.

16. Zhao J, Jia J, Qian Y, Zhong L, Wang J and Cai Y. COVID-19 in Shanghai: IPC Policy Exploration in Support of Work Resumption Through System Dynamics Modeling. Risk Manag Healthc Policy. 2020;13:1951-1963.

17. Mahikul W, Chotsiri P, Ploddi K and Pan-ngum W. Evaluating the Impact of Intervention Strategies on the First Wave and Predicting the Second Wave of COVID-19 in Thailand: A Mathematical Modeling Study. Biology (Basel). 2021;10:80.

18. Gupta M, Mohanta S S, Rao A, Parameswaran G G, Agarwal M, Arora M, et al. Transmission dynamics of the COVID-19 epidemic in India and modeling optimal lockdown exit strategies. Int J Infect Dis. 2021;103:579-589.

19. Aldila D, Khoshnaw S H A, Safitri E, Anwar Y R, Bakry A R Q, Samiadji B M, et al. A mathematical study on the spread of COVID-19 considering social distancing and rapid assessment: The case of Jakarta, Indonesia. Chaos Solitons Fractals. 2020;139:110042.

20. Okuonghae D and Omame A. Analysis of a mathematical model for COVID-19 population dynamics in Lagos, Nigeria. Chaos Solitons Fractals. 2020;139:110032.

21. Barbarossa M V, Fuhrmann J, Meinke J H, Krieg S, Varma H V, Castelletti N, et al. Modeling the spread of COVID-19 in Germany: Early assessment and possible scenarios. Plos One. 2020;15:e0238559.

22. Shen M, Zu J, Fairley C K, Pagán J A, Ferket B, Liu B, et al. Effects of New York's Executive Order on Face Mask Use on COVID-19 Infections and Mortality: A Modeling Study. J Urban Health. 2021;98:197-204.

23. Sun J, Chen X, Zhang Z, Lai S, Zhao B, Liu H, et al. Forecasting the long-term trend of COVID-19 epidemic using a dynamic model. Sci Rep. 2020;10:21122.

24. Zhang X S, Vynnycky E, Charlett A, De Angelis D, Chen Z and Liu W. Transmission dynamics and control measures of COVID-19 outbreak in China: a modelling study. Sci Rep. 2021;11:2652.

25. Asamoah J K K, Jin Z, Sun G-Q, Seidu B, Yankson E, Abidemi A, et al. Sensitivity assessment and optimal economic evaluation of a new COVID-19 compartmental epidemic model with control interventions. Chaos Solitons Fractals. 2021;146:110885.

26. Mushayabasa S, Ngarakana-Gwasira E T and Mushanyu J. On the role of governmental action and individual reaction on COVID-19 dynamics in South Africa: A mathematical modelling study. Inform Med Unlocked. 2020;20:100387.

27. Choi W and Shim E. Optimal strategies for social distancing and testing to control COVID-19. J Theor Biol. 2021;512:110568.

28. Chiu W A, Fischer R and Ndeffo-Mbah M L. State-level needs for social distancing and contact tracing to contain COVID-19 in the United States. Nat Hum Behav. 2020;4:1080-1090.

29. Ivorra B, Ferrández M R, Vela-Pérez M and Ramos A M. Mathematical modeling of the spread of the coronavirus disease 2019 (COVID-19) taking into account the undetected infections. The case of China. Commun Nonlinear Sci Numer Simul. 2020;88:105303.

30. Ramos A M, Ferrández M R, Vela-Pérez M, Kubik A B and Ivorra B. A simple but complex enough θ -SIR type model to be used with COVID-19 real data. Application to the case of Italy. Physica D. 2021;421:132839.

31. Aldila D, Samiadji B M, Simorangkir G M, Khosnaw S H A and Shahzad M. Impact of early detection and vaccination strategy in COVID-19 eradication program in Jakarta, Indonesia. BMC Res Notes. 2021;14:132.

32. Balsa C, Lopes I, Guarda T and Rufino J. Computational simulation of the COVID-19 epidemic with the SEIR stochastic model. Comput Math Organ Theory. 2021;1-19.

33. Iboi E A, Ngonghala C N and Gumel A B. Will an imperfect vaccine curtail the COVID-19 pandemic in the U.S.? Infect Dis Model. 2020;5:510-524.

34. Ghostine R, Gharamti M, Hassrouny S and Hoteit I. An Extended SEIR Model with Vaccination for Forecasting the COVID-19 Pandemic in Saudi Arabia Using an Ensemble Kalman Filter. Mathematics. 2021;9:636.

35. Dashtbali M and Mirzaie M. A compartmental model that predicts the effect of social distancing and vaccination on controlling COVID-19. Sci Rep. 2021;11:8191.
