# Supplementary material for: Compartmental structures used in modeling COVID-19: a scoping review
Source: Infect Dis Poverty. 2022 Jun 21;11:72. doi: 10.1186/s40249-022-01001-y (PMC9209832; doi:10.1186/s40249-022-01001-y)
Supplement: Supplementary file 5 — Additional file 5. Compartmental structures of agent-based models for COVID-19. [file 40249_2022_1001_MOESM5_ESM.docx]

Table Compartmental structures of agent-based models for COVID-19.

| Compartmental structure | Interpretation | Reference |
| --- | --- | --- |
| SEAI_m_I_s_I_c_RD | susceptible (S), exposed (E), asymptomatic (A), mild (I_m_), severe (I_s_), critical (I_c_), recovered (R), dead (D） | [[1](#_ENREF_1)] |
| SUPCR | susceptible (S), undetected infected (U), positive infections detected from testing (P), contacts traced (C), recovered (R) | [[2](#_ENREF_2)] |
| SL_a_L_s_P_s_I_a_I_s_HH_ICU_R | susceptible (S), latent asymptomatic (L_a_), latent symptomatic (L_s_), pre-symptomatic (P_s_), asymptomatic (I_a_), symptomatic (I_s_), hospitalized (H), hospitalized in intensive care (H_ICU_), recovered (R) | [[3](#_ENREF_3)] |
| SEI_p_I_a_I_s_I_h_RD | susceptible (S), exposed (E), pre-symptomatic (I_p_), asymptomatic (I_a_), symptomatic (I_s_), hospitalized (I_h_), recovered (R), dead(D) | [[4](#_ENREF_4)] |
| SEI_a_I_s_I_c_RMD | susceptible (S), exposed (E), pre-symptomatic (I_a_), seriously-infected (I_s_), critically-infected (I_c_), recovered (R), immune (M), dead (D) | [[5](#_ENREF_5)] |
| SEI_a_I_b_I_s_I_c_RD | susceptible (S), exposed-incubation(E), unknown mild infected (I_a_), known mild infected (I_b_), severe infected (I_s_), critical infected (I_c_), recovered (R), dead (D) | [[6](#_ENREF_6)] |
| SEI_p_I_a_I_m_I_s_I_c_RD | susceptible (S), exposed (E), pre-symptomatic (I_p_), asymptomatic (I_a_), mild (I_m_), severe (I_s_), critical (I_c_), recovered (R), dead (D) | [[7](#_ENREF_7), [8](#_ENREF_8)] |
| SII_a_I_s_HH_ICU_RD | susceptible (S), incubation (I), asymptomatic (I_a_), symptomatic (I_s_), hospitalized (H), ICU (H_ICU_), recovered (R), dead (D) | [[9](#_ENREF_9)] |
| SEPAII_q_R | susceptible (S), exposed (E), pre-symptomatic (P), asymptomatic (A), symptomatic (I), isolated symptomatic (I_q_), recovered (R) | [[10](#_ENREF_10)] |
| SEIDRTH | susceptible (S), exposed (E), symptomatic (I), dead (D), recovered (R), testing in a test car (T_C_), testing in a hospital (T_H_), normal hospitalization (H_N_), hospitalization in an intensive care unit (H_ICU_) | [[11](#_ENREF_11)] |
| SEACH_1_H_2_D_1_D_2_RM | susceptible (S), exposed (E), asymptomatic (A), symptomatic, will recover (C), symptomatic and will recover, not yet hospitalized (H_1_), hospitalized and will recover (H_2_), symptomatic, not yet hospitalized (D_1_), hospitalized and will die (D_2_), recovered (R), dead (M) | [[12](#_ENREF_12)] |

**Reference**

1. Pham Q D, Stuart R M, Nguyen T V, Luong Q C, Tran Q D, Pham T Q, et al. Estimating and mitigating the risk of COVID-19 epidemic rebound associated with reopening of international borders in Vietnam: a modelling study. Lancet Glob Health. 2021;9:e916-e924.

2. Mukherjee U K, Bose S, Ivanov A, Souyris S, Seshadri S, Sridhar P, et al. Evaluation of reopening strategies for educational institutions during COVID-19 through agent based simulation. Sci Rep. 2021;11:20667.

3. Aleta A, Martín-Corral D, Pastore Y P A, Ajelli M, Litvinova M, Chinazzi M, et al. Modelling the impact of testing, contact tracing and household quarantine on second waves of COVID-19. Nat Hum Behav. 2020;4:964-971.

4. Patel M D, Rosenstrom E, Ivy J S, Mayorga M E, Keskinocak P, Boyce R M, et al. The Joint Impact of COVID-19 Vaccination and Non-Pharmaceutical Interventions on Infections, Hospitalizations, and Mortality: An Agent-Based Simulation. medRxiv. 2021. doi:10.1101/2020.12.30.20248888.

5. Gomez J, Prieto J, Leon E and Rodriguez A. INFEKTA-An agent-based model for transmission of infectious diseases: The COVID-19 case in Bogota, Colombia. Plos One. 2021;16:e0245787.

6. Alagoz O, Sethi A K, Patterson B W, Churpek M and Safdar N. Effect of Timing of and Adherence to Social Distancing Measures on COVID-19 Burden in the United States A Simulation Modeling Approach. Ann Intern Med. 2021;174:50-57.

7. Panovska-Griffiths J, Kerr C C, Stuart R M, Mistry D, Klein D J, Viner R M, et al. Determining the optimal strategy for reopening schools, the impact of test and trace interventions, and the risk of occurrence of a second COVID-19 epidemic wave in the UK: a modelling study. Lancet Child Adolesc Health. 2020;4:817-827.

8. Flaxman S, Mishra S, Gandy A, Unwin H J T, Mellan T A, Coupland H, et al. Estimating the effects of non-pharmaceutical interventions on COVID-19 in Europe. Nature. 2020;584:257-261.

9. Lima L L and Atman A P F. Impact of mobility restriction in COVID-19 superspreading events using agent-based model. Plos One. 2021;16:e0248708.

10. Asgary A, Cojocaru M G, Najafabadi M M and Wu J. Simulating preventative testing of SARS-CoV-2 in schools: policy implications. Bmc Public Health. 2021;21:125.

11. Truszkowska A, Behring B, Hasanyan J, Zino L, Butail S, Caroppo E, et al. High-Resolution Agent-Based Modeling of COVID-19 Spreading in a Small Town. Adv Theory Simul. 2021;4:2000277.

12. Head J R, Andrejko K, Cheng Q, Collender P A, Phillips S, Boser A, et al. The effect of school closures and reopening strategies on COVID-19 infection dynamics in the San Francisco Bay Area: a cross-sectional survey and modeling analysis. medRxiv. 2020. doi:10.1101/2020.08.06.20169797.
